# Shared Multimodal Input Through Social Coordination: Infants With Monolingual and Bilingual Learning Experiences

**DOI:** 10.3389/fpsyg.2022.745904

**Published:** 2022-04-19

**Authors:** Lichao Sun, Christina D. Griep, Hanako Yoshida

**Affiliations:** Department of Psychology, University of Houston, Houston, TX, United States

**Keywords:** head-mounted eye tracker, object play, bilingual culture, infant attention, multimodal input

## Abstract

A growing number of children in the United States are exposed to multiple languages at home from birth. However, relatively little is known about the early process of word learning—how words are mapped to the referent in their child-centered learning experiences. The present study defined parental input operationally as the integrated and multimodal learning experiences as an infant engages with his/her parent in an interactive play session with objects. By using a head-mounted eye tracking device, we recorded visual scenes from the infant’s point of view, along with the parent’s social input with respect to gaze, labeling, and actions of object handling. Fifty-one infants and toddlers (aged 6–18 months) from an English monolingual or a diverse bilingual household were recruited to observe the early multimodal learning experiences in an object play session. Despite that monolingual parents spoke more and labeled more frequently relative to bilingual parents, infants from both language groups benefit from a comparable amount of socially coordinated experiences where parents name the object while the object is looked at by the infant. Also, a sequential path analysis reveals multiple social coordinated pathways that facilitate infant object looking. Specifically, young children’s attention to the referent objects is directly influenced by parent’s object handling. These findings point to the new approach to early language input and how multimodal learning experiences are coordinated socially for young children growing up with monolingual and bilingual learning contexts.

## Introduction

Approximately one in four children in the United States is growing up in a multilingual learning context, where the child is exposed to multiple languages at home from birth (U.S. Census Bureau, 2015). Such a language background is often considered as one’s individual characteristic (e.g., monolingual vs. bilingual) and linked to developmental consequences. The differential outcomes between monolingual and bilingual children have been documented across a variety of domains, including language outcomes (Allman, 2005; Hammer et al., 2007; Bialystok et al., 2010; Thordardottir, 2011; Hoff and Core, 2013; De Houwer et al., 2014, 2018; Hoff et al., 2014), cognitive outcomes (Carlson and Meltzoff, 2008; Kovács and Mehler, 2009a; Bialystok and Craik, 2010; Hernández et al., 2010; Brito and Barr, 2014; Bialystok, 2015; Arredondo et al., 2017; D’Souza et al., 2020), and academic outcomes (Lindholm and Aclan, 1991; Kovelman et al., 2008; Rodríguez et al., 2014; Spitzer, 2016; Festman and Schwieter, 2019). Furthermore, the bilingual literature often focuses on children who are mature enough to demonstrate their use of multiple languages. While this research reveals a clear impact of learning multiple languages, we still do not know the origin of the observed differences—how hearing and learning multiple languages initially may differ from hearing and learning only one language.

Recent studies indicate that the impacts of infants being exposed to multiple languages emerge as early as 6 months of age (Kovács and Mehler, 2009a,b; Brito and Barr, 2014; Singh et al., 2015; Comishen et al., 2019; Arredondo et al., 2022). To separate and discriminate languages, infants who are simultaneously learning dual language from birth have shown advances in phonetic sensitivities and initiated differences in speech perception and word recognition skills (Werker and Byers-Heinlein, 2008; Werker et al., 2009; Sebastián-Gallés et al., 2012; Singh et al., 2018; Höhle et al., 2020; Kalashnikova and Carreiras, 2022). Other observational studies also provide detailed characteristics of *linguistic input* in early childhood and reveal the variations in the language learning context with respect to the amount of exposure in each language, the similarity between two languages, the speech type, parent’s language proficiency, and so forth (De Houwer, 2007; Thordardottir, 2011; Ramírez-Esparza et al., 2014, 2017; De Houwer et al., 2018; Carbajal and Peperkamp, 2020; Orena et al., 2020).

However, the existing work has been limited to describing children’s verbal input without characterizing the interactive learning experiences from the child’s perspective. Language learning depends not only on the amount of verbal exposure in a specific language environment; acquiring a new word requires the child to pay attention to what the parent is referring to. Despite the prevalence of early dual-language exposure at home and its potential broad impacts on developmental milestones, surprisingly little research has been conducted on the foundations of bilingual language learning, *viz., the language learning processes* that an infant may face many uncertain referents when hearing a word that she/he does not yet know. The present study aims to characterize the child-centered perceptual experiences of how the heard words are linked to their visual perception in parent–child social interactions to fully capture the child-centered learning experiences.

As a first step toward understanding early child language experiences, the present study focuses on moment-to-moment multimodal experiences—looking and hearing—in an interactive social environment. Using head-mounted eye-tracking camera recordings, we examine differences and similarities in *multisensory input* between infants growing up in monolingual and multilingual home contexts. We specifically examine (1) parent’s social input, (2) infant’s looking behaviors, and (3) the social coordination between parent and infant during an object play as a function of language learning context (monolingual vs. bilingual home environments). In addition to the documented differences in the amount of parental verbal input, we also expect that infants from both monolingual and bilingual households may experience similar or different word–referent mapping moments, which may serve as the foundation for later word learning.

### Bilingual Language Development

In the domain of language development, there is an established line of work focusing on language input—how daily bilingual exposure influences linguistic experiences (De Houwer, 2007; Rowe, 2012; Cartmill et al., 2013; Ramírez-Esparza et al., 2014, 2017; Carroll, 2017; De Houwer et al., 2018). Researchers typically use language diaries to record numerous conversations across a variety of contexts (e.g., Huttenlocher et al., 2010; Place and Hoff, 2011; Rowe, 2012; Gilkerson et al., 2017; Carbajal and Peperkamp, 2020) and/or count the number of words and assess the word types, sentence complexity, and contexts in which words and sentences are used by parental questionnaires (e.g., De Houwer, 2007, 2011; Scheele et al., 2010; Byers-Heinlein, 2013; Unsworth et al., 2019).

These rigorous studies of early linguistic experiences are often conducted in the context of complex sociocultural backgrounds in which families use dual or even triple languages at home. Children who are exposed to two languages from birth have been shown to learn fewer words in the dominant language and to have different growth trajectories of receptive and expressive vocabulary than their monolingual counterparts in preschool and school ages (Bialystok et al., 2010; Poulin-Dubois et al., 2013; Hoff et al., 2014; Hoff and Ribbot, 2017). The demonstrated variability in language growth in children from diverse language learning environments have been documented with and without consideration of socioeconomic status (SES; De Houwer, 2007; Cartmill et al., 2013; MacLeod et al., 2013; Gilkerson et al., 2017; Hoff and Ribbot, 2017; Ramírez-Esparza et al., 2017; Masek et al., 2021). Moreover, some argue that bilingual learning environments are often associated with relatively limited exposure in each language (e.g., De Houwer, 2007; Thordardottir, 2011; Hoff et al., 2012) as well as less diverse and sophisticated input (e.g., Rowe, 2012; Place and Hoff, 2016; Unsworth et al., 2019).

However, understanding word acquisition requires more than characterizing the linguistic input only. Learners may come with biases and intentions as children actively engage in their social world, and thus, they distort the regularities and carve the input systematically. Recent literature indicates the importance of quality learning from the child’s perspective, and these studies focus on the social coordination generated by the child and the parent together, such as object labeling while seeing the object simultaneously (e.g., Yu and Smith, 2012; Pereira et al., 2014). Such coordinated experience predicts the child’s later vocabulary learning more strongly than the amount of verbal input alone (Sun and Yoshida, 2018, 2019). The present study aims to precisely record word–referent mapping experiences to assess the quality of word learning at a new level.

### Sociocultural Impacts on Bilingual Language Development

Language is an extension of sociocultural practices, and research has demonstrated that language development is impacted by one’s sociocultural context (Tamis-LeMonda and Song, 2013; Kandhadai et al., 2014; Kramsch, 2014). One domain in which sociocultural practices impact early language development is the communicative pattern between the parent and the child (Tamis-LeMonda et al., 1992, 2013; Ramírez-Esparza et al., 2017). Parents from various sociocultural backgrounds may respond to their children differently in terms of the rate of utterances, the speech style (adult-directed vs. infant-directed), as well as the composition of language (noun vs. verb; Tardif, 1996; Shneidman and Goldin-Meadow, 2012; Tamis-LeMonda et al., 2013; Farran et al., 2016). While bilingual research often compares monolingual and bilingual groups within a specific cultural background (e.g., Barac and Bialystok, 2012), Tran et al. (2019) examined the effects of bilingualism across different countries and cultures (i.e., Vietnam, Argentina, and the United States) and revealed a consistent impact of bilingualism on children’s attention and executive control. This finding points to the existence of unique characteristics of bilingual learning experiences that are shared across a variety of cultural backgrounds.

Scaling down from the macro sociocultural aspects of a specific cultural background, the individual child’s language development begins in a *social context through immediate interaction with parents*. Parent scaffolding plays a key role in navigating infant attention toward the region of interests (ROIs, e.g., Butterworth and Cochran, 1980; Bakeman and Adamson, 1984; Tamis-LeMonda et al., 2014). For instance, infants appear to shift attention to the general direction of the caregiver’s eye gaze from the age of 2 months (Butterworth and Cochran, 1980; Gredebäck et al., 2010; Simpson et al., 2020), and they subsequently exhibit precise gaze shifting to the targeted object around 1 year of age (Brooks and Meltzoff, 2002, 2005). The ability to successfully shift attention to the target of interest establishes the foundation of social learning and increases the learning efficiency that quickly maps the referent words to the seen objects (Baldwin, 1993; Yu and Smith, 2012; Tenenbaum et al., 2014). Developmental researchers have studied infant object looking with respect to several aspects of parental social references, including parent’s gaze (Brooks and Meltzoff, 2002; Caron et al., 2002), verbal phrases and labels (Flom and Pick, 2003; Fulkerson and Haaf, 2003), hand actions (Yu and Smith, 2013, 2017; Deák et al., 2014; Burling and Yoshida, 2019), and the combined use of multimodal references by the parents (Gogate et al., 2006; Tamis-LeMonda et al., 2013; Deák et al., 2018; Suarez-Rivera et al., 2019). The diverse social inputs serve as the perceptual foundation of word learning and as a basis for establishing the social coordination between infants and parents that is essential for sharing a common focus of attention.

### Multimodal Experiences for Language Learning

A fundamental process of early language acquisition involves multimodal learning experiences—children constantly hear words while *seeing* the referents, either the objects or the people that are relevant to the referents. This conjunction of sound and sight serves as a building block for the initial process of language learning—mapping a word to its meaning. The ability to form word–referent associations is universal across monolingual and bilingual environments (Byers-Heinlein et al., 2013). One line of research has focused on the visual side of word learning and has provided information about the infant’s moment-to-moment gaze behaviors during the parent–infant object play. These studies indicate that there are powerful input structures in everyday experiences with parents that create developmentally appropriate attention-directing links between heard words and seen referents/objects (Gogate et al., 2006; Smith and Yu, 2008; Koterba and Iverson, 2009). Studies using head-mounted camera devices to record the infant centered views during object play found that infants often have a clear view of the object (Yoshida and Smith, 2008; Yoshida and Fausey, 2019), and such clear viewing of an object is often accompanied by parental references (e.g., Yu and Smith, 2017; Burling and Yoshida, 2019; Yu et al., 2019). When the parent names the object at those socially synchronized moments, infants are more likely to learn the name of the object (Gogate et al., 2006; Yu and Smith, 2012; Pereira et al., 2014; Yoshida and Fausey, 2019). These eye-tracking studies of child-centered input make it clear that not only the number of words that an infant hears during the interaction is important, but also the contingency between infant’s visual experiences and appropriate linguistic input matters for effective word learning. However, all the existing evidence on early multimodal learning experiences was concluded from Caucasian, middle class, and monolingual families only. Yet the multimodal experience has not been explored in a dual-language learning context. Considering their differences in verbal input, attention processes, and various language learning trajectories in each language, it is essential to investigate whether bilingual children experience similar or different socially coordinated moments, as do their monolingual peers.

The present study examined the microstructure of multimodal learning experience in the infant’s interactive object play with his/her parents and attempted to identify the similarities and differences between parent–child dyads having a monolingual versus a bilingual learning context at home. The present study focuses on (1) the parent’s social input, as reflected in looking patterns as well as verbal input, (2) the infant’s looking behaviors, and (3) the social coordination between parent and infant during the play session. We expected to see the documented differences in linguistic input, but we also anticipated that participants from both monolingual and bilingual backgrounds would exhibit similar or different word–referent mapping experiences according to the parent’s input in the instances of social coordination.

## Materials and Methods

### Participants

Fifty-one parent–infant dyads, which included 6- to 18-month-old infants and toddlers (Mean age = 11.1 months, SD = 3.9), were recruited from the Greater Houston area. An additional eight dyads were recruited but not included due to incomplete data collection associated with infant fussiness, technical failure, or inadequate recording quality. All parent participants gave their informed consent for inclusion before they participated in the study. A small gift bundle, including a gift card, a museum family pass, an infant-sized t-shirt, and a stuffed animal, was provided to every parent–infant dyad. The study protocol was approved by the Institutional Review Board in the local research community. Parents completed the informed consent regarding their participation prior to the lab visit. The ethnic backgrounds represented were as follows: Caucasian (33%), Hispanic or Latino (27%); African American (8%); Asian (8%); two or more races (16%), and unidentified (8%). All the infants were full-term and typically developing with no speech/hearing/vision issues.

Considering the high level of linguistic diversity in the Greater Houston areas, we categorized the dyad’s language learning environment into bilingual or monolingual groups according to the home language usage reported by parents. Infants were considered to live in a consistent bilingual language learning environment when (1) they were exposed to a dual-language home environment since birth, and (2) parents spent over 20% of the time using the minority language at home.

There were 22 dyads from English monolingual households (Mean age = 11.2 months, SD = 4.3) and 29 dyads from diverse bilingual households (Mean Age = 11.1 months, SD = 3.6). Infants in the bilingual group were exposed to English and other languages as follows: Spanish (19), Vietnamese (3), Chinese (2), German (1), Tamil (1), Krio (1), and unidentified (2). We compared the demographic variables between groups and found no significant differences in (1) infant’s age [*t*(41) = −0.06, *p* = 0.954], (2) birth order (*p* = 0.870, Fisher’s exact test), and (3) parental education level, *p* = 0.37 by Fisher’s exact test. Find the detailed demographic distributions in the Supplementary Material.

### Procedures

All the participating dyads completed an object play session in the lab with the same procedure. Participants were led to the experimental rooms and asked to sit across from each other at a 60 × 60 × 40 cm table, which was used as a surface for jointly interacting with the objects. The experimenter helped both parent and infant put on the head-camera gears. After the research assistants completed the calibration procedure, the parent was provided with a container of attractive toys and instructed to freely play with the infant. The box consisted of eight toys: a bunny, a cookie, a cup, a car, a bear, a jar, a carrot, and a basket.

To structure the play session, parents were encouraged to freely use any of the toys as they played with their infant around the theme words. The target words included four nouns (i.e., bunny, car, cookie, and cup) and four verbs (i.e., open, put, eat, and drink). These words and objects were selected based on the infant’s early learned words on the MacArthur Communicative Development Inventories (MCDI; Fenson et al., 2000). An audio instruction provided cues for parents as to which target word constructs the play. For the bilingual parents, we asked the parent’s language preference prior to the play session and provided the instruction in languages other than English given their needs. Three of the bilingual parents chose the Spanish instruction and used Spanish target words throughout the play, whereas the rest of the sample followed the English instruction. The main goal of the play session was not to ask parents to teach or demonstrate the target words but to provide an interactive context in which parents and infants can jointly play with the objects together. Therefore, bilingual parents were encouraged to use the language they were most comfortable with or switch to using dual language and act naturally with their infants the same way they would at home. The play session consisted of eight 40-s-long trials, for a total of 5 min and 20 s.

### Equipment

Watec (WAT-230A) miniature color cameras with supplementary eye trackers were used for recording the object play session. The head-mounted camera provides dynamic visual information from a first-person view (e.g., Yoshida and Smith, 2008; Smith et al., 2011; also see a review from Smith et al., 2015). This camera moves with the participant’s head to indicate what is in the participant’s view from moment to moment. Previous studies using similar head-mounted cameras compared head versus eye direction in toddlers and found a 90% correspondence between the directions of head movement and eye gazes (Yoshida and Smith, 2008).

In addition to the head-mounted camera system, an eye tracker was used to specify the focus of visual attention in each frame of the egocentric views. Correspondence between the images from the head-mounted camera and eye tracker was achieved using a manual calibration procedure. We used a 60 × 40 cm board with nine spatially distributed stickers. Before and after the play session, research assistants would point to each sticker using a salient, jingling fish toy to attract the infant’s attention. The same calibration procedure was repeated for the parent as well. The location of eye gaze on the scenery image from the egocentric view was estimated by the Yarbus software. A minimum calibration correlation of 0.9 between the camera and the eye-tracker images was obtained.

Two additional digital video cameras and an audio recorder were mounted on the wall and the ceiling to capture an overall view of the scene in which the play session took place. All the videos were recorded at a rate of 30 frames per second and were synchronized by Adobe Premiere with the same sampling rate. On average, every parent–infant dyad had 8,653 frames (SD: 439) recorded during the play session and used for analysis. The inaccessible frames include eye blinks and interruptions due to camera adjustment and child fussiness.

### Parental Questionnaires

After the play session, parents were asked to complete the MacArthur SES form and the MCDI checklist. The MacArthur SES form included demographic measures with respect to parental education level, occupation, family size and relationship, annual household income, health conditions, and so forth. In addition, we added a series of questions to specify the participant’s language status, including questions on (1) bilingualism (i.e., “Is your child exposed to a language other than English?”), (2) language type (i.e., “What language(s)?” and “By whom?”), (3) the age of acquisition (“Since what age (in months)?”), and (4) each language usage (i.e., “How many days per week?” and “How many hours per day?”). Bilingual’s daily language exposure in L1 and L2 are summarized in Table 1.

**Table 1:** Bilingual’s daily language exposure in L1 and L2.

Variables	Mean	Standard deviation
The days per week in L1	6.9	0.4
The hours per day in L1	18.5	6.8
Age of acquisition in L1	0.6	1.3
The days per week in L2	6.3	1.3
The hours per day in L2	7.0	7.4
Age of acquisition in L2	2.2	3.6

In addition, parents completed the MCDI checklist of words and gestures, which has been widely used for assessing communication skills in infants and toddlers. Construct reliability and validity of the infant’s form reached Cronbach’s coefficient alpha of 0.97 and 0.90, respectively (Fenson et al., 2000). Table 2 presents the infant’s receptive and productive vocabulary by language groups. In specific, infants in both language groups had comparable numbers of phrases understood, words understood, and known target words before participating in the study.

**Table 2:** Monolingual and bilingual infant’s MCDI scores.

Measures	Monolingual mean (std)	Bilingual mean (std)	*t*-value	*p*-value
The number of words understand	64.5 *(78.5)*	39.1 *(29.4)*	1.35	0.191
The number of words understand and say	20.0 *(40.6)*	4.5 *(10.1)*	1.71	0.102
The number of phrases understand	12.4 *(10.1)*	11.4 *(8.5)*	0.36	0.724
The number of target words understand (total = 8)	3.1 (3.1)	2.5 (2.3)	0.61	0.543

*The italics values refer to the standard deviation*.

### Behavioral Annotation

Each dyad’s videos, including the views from parent, infant, wall, and ceiling, were synchronized by Adobe Premiere and further imported into the Datavyu software. Two well-trained coders, blind to the purpose of the study, annotated the behavioral variables for each parent–infant dyad. Table 3 presents all the annotated behaviors, including infant’s and parent’s looking pattern, parental referential input, and the coordinated attention between infant’s object looking and multimodal social references by parents.

**Table 3:** Definitions of behavioral measures.

Measures	Definition	Example
Parent’s attention pattern	Gaze allocation on the four ROIs	Attention on child’s face, parent’s hands, child’s hands, or target objects
*Parent’s phrases*		
(1) All phrase use	Any phrase use during the play session	“Look at the bunny” “What is that?”
(2) Phrases containing relevant labels	Phrase containing at least one labels on any of the target object	“Do you like the **bear**?”
(3) Phrases containing irrelevant labels	Phrase containing with no relevant labels	“Yummy!” “Look at this.” “Mira! (Look in Spanish)”
(4) Target labels	Eight target labels	Bunny, eat, cookie, car, bear, put, drink, open
Infant’s attention pattern	Gaze allocation on the four ROIs	Attention on parent’s face, parent’s hands, child’s hands, or target objects
All phrase use while the infant looked at an object	Any phrase when the infant looked at the target object	
Phrase containing the target label while the infant looked at the target object	Phrase containing at least one relevant label when the infant looked at the target object	
Optimal naming moment	Phrase containing at least one relevant label when the infant looked at the target object and over 70% of the object has been captured from the child’s view	
Joint attention	Shared attention between parent and infant on the same target object	
Sustained attention	Infant attention on the target object and maintained over 2 s long	

First, both the infant’s and parent’s looking patterns were annotated from their egocentric views captured by the head-mounted cameras. The number of frames was counted to estimate both the frequency and duration of an individual’s attention with respect to four ROIs: (1) target object, (2) parent’s face, (3) parent’s hands, and (4) infant’s hands. We chose these four ROIs because the social partner’s face and hands are shown as the most visually accessible areas in the infant’s point of views, according to observation studies using the head-camera devices (Yoshida and Smith, 2008; Yu and Smith, 2013; Deák et al., 2018).

Specifically, the following analyses primarily focused on the instances of infant object looking. Reliability was measured by randomly selecting 25% of the frames for each dyad and assessing inter-rater coding agreement for the infant’s viewing behaviors. The inter-coder reliability of the infant’s gaze was an average of 83%, as assessed by Cohen’s kappa of 0.73 (ranging from 0.51 to 0.91). The obtained reliability rate falls into the reliability criteria applied in other eye-tracking studies (84% for Yoshida et al., 2020, 82–95% in Yu and Smith, 2017; 83% for Chang et al., 2016).

Moreover, parent’s phrases and the use of target words were transcribed and counted. A total of 5,122 phrases were added in the following analyses. Of all the phrases, 22.8% of phrases in the bilingual sample (*N* = 2,591) were translated from other languages by research assistants who are fluent in the respective language. Bilingual parents’ language usages and the number of translated phrases can be found in the Supplementary Material. The reliability was achieved by randomly selecting 25% of the total phrases by dyad and met the inter-coder agreement of 95% or above. Considering the majority of infants vocalized only during the play session, their verbal responses were excluded in the following analyses.

**Figure 1:**
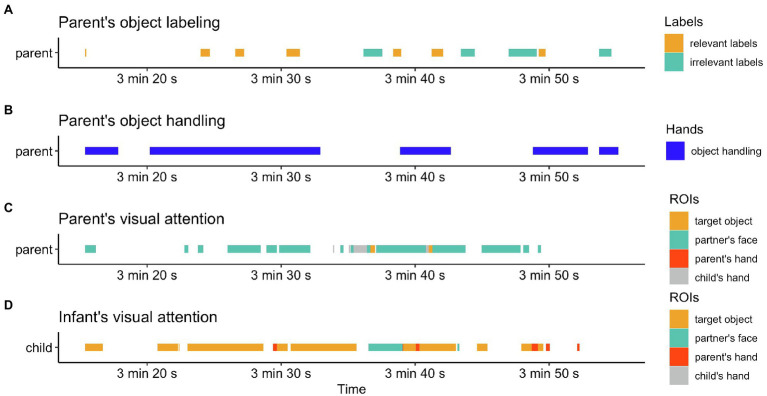
The multimodal behavioral annotation of a video clip of a parent–infant play session.

In addition to the parent’s attention patterns and phrases, object manipulation was also annotated from one’s egocentric views. The wall and ceiling views were also treated as supplementary references when the parent’s object handling was not easily interpreted or not captured by the head-mounted cameras. Each of the parental references was manually annotated separately and then time-stamped together in correspondence with the timeline of the play session (see Figure 1).

### Analytical Plan

Table 4 provides the summary statistics, by language groups, for various measures of parent and infant behaviors within ROIs. Considering the intercorrelation among the multiple dependent variables and potential variations among dyads, a series of multivariate analyses of variance with age and SES as covariates (MANCOVA) were selected. Instead of a separate univariate analysis on each dependent variable, MANCOVA reduces potential inflation on Type I error. SES accounted for both annual household income and parental highest degrees. Specifically, parents from the middle class met one of the following criteria, and the higher class met both criteria: (1) annual household income of $47,000 or greater (i.e., the median annual household income in Houston; U.S. Census Bureau, 2020), and (2) at least one parent with a bachelor’s or higher degree. The dependent measures of the comparison models included (1) parent responsiveness in terms of looking patterns and verbal input, (2) infant’s looking behaviors, and (3) the social coordination between parent and infant during the play session. For both parent’s and infant’s looking patterns, we measured the frequency and duration of gaze in each of the four target ROIs. Parental phrases were categorized into phrases with relevant or irrelevant labels, depending on whether any of the target words was used. The frequency of relevant labels was also counted independently.

**Table 4:** Frequency and duration (s) of observed behaviors, averaged across infant participants within the bilingual and monolingual language groups.

Group comparisons	Measures	Bilingual mean (std)	Monolingual mean (std)	*t*-value	*p*-value
Parent object handling	Time duration	260.0 (*41.7*)	266.4 (*44.0*)	−0.53	*0.602*
	Frequency	86.6 (*36.7*)	80.9 (*22.0*)	0.70	*0.490*
Infant object handling	Time duration	188.8 (*89.6*)	177.7 (*97.7*)	0.42	*0.680*
	Frequency	61.2 (*44.6*)	44.2 (*26.5*)	1.69	0.097
Parent’s look at the target objects	Time duration	66.4 (*41.0*)	71.6 (*41.8*)	−0.45	*0.658*
	Frequency	197.9 (*121.5*)	231.9 (*145.5*)	−0.89	*0.381*
Parent’s look at the infant’s face	Time duration	110.5 (*60.1*)	102.1 (*52.8*)	0.53	*0.596*
	Frequency	188.4 (*113.9*)	212.5 (*182.9*)	−0.54	*0.590*
Parent’s look at the infant’s hands	Time duration	22.5 (*17.3*)	19.8 (*12.6*)	0.63	*0.529*
	Frequency	95.1 (*80.6*)	86.0 (*52.8*)	0.48	*0.631*
Parent’s look at their own hands	Time duration	12.8 (*11.7*)	12.6 (*11.8*)	0.05	*0.960*
	Frequency	56.5 (*52.3*)	69.2 (*78.9*)	−0.66	*0.516*
Infant’s look at the target objects	Time duration	150.5 (*61.8*)	148.3 (*45.4*)	0.15	*0.884*
	Frequency	120.3 (*54.1*)	156.2 (*67.2*)	−2.05	***0.047***
Infant’s look at the parent’s face	Time duration	23.2 (*18.0*)	21.3 (*13.8*)	0.42	*0.675*
	Frequency	26.7 (*24.4*)	30.2 (*16.5*)	−0.62	*0.541*
Infant’s look at their own hands	Time duration	2.6 (*4.1*)	1.6 (*2.9*)	0.94	*0.351*
	Frequency	6.7 (*8.0*)	6.9 (*12.5*)	−0.05	*0.964*
Infant’s look at the parent’s hands	Time duration	30.8 (*19.2*)	37.7 (*25.4*)	−1.08	*0.289*
	Frequency	66.8 (*38.8*)	88.3 (*47.7*)	−1.72	*0.092*
Parent’s phrase	Time duration	132.8 (*53.2*)	181.4 (*57.7*)	−3.08	***0.004***
	Frequency	89.3 (*37.6*)	115.0 (*27.8*)	−2.81	***0.007***
Parent’s phrase with relevant labels	Time duration	78.5 (*40.1*)	106.2 (*38.4*)	−2.50	***0.016***
	Frequency	51.3 (*26.3*)	66.5 (*21.0*)	−2.29	***0.026***
Parent’s phrase with irrelevant labels	Time duration	54.4 (*34.2*)	75.2 (*42.8*)	−1.88	*0.068*
	Frequency	38.0 (*24.0*)	48.5 (*22.1*)	−1.62	*0.112*
The number of object labeling	Frequency	56.9 (*29.5*)	80.4 (*24.7*)	−3.09	***0.003***
Parent’s phrase while the child looked at the target objects	Time duration	69.6 (*49.3*)	86.0 (*35.4*)	−1.38	*0.174*
	Frequency	81.5 (*38.4*)	128.2 (*51.4*)	−3.57	***0.001***
Parent’s object labeling while the infant looked at the target object	Time duration	42.6 (*31.4*)	53.0 (*22.4*)	−1.38	*0.173*
	Frequency	50.0 (*28.8*)	75.8 (*25.3*)	−3.39	***0.001***
Optimal naming moment	Time duration	22.2 (*17.4*)	29.3 (*17.5*)	−1.43	*0.159*
	Frequency	26.2 (*17.2*)	42.5 (*24.2*)	−2.69	***0.011***
Joint attention toward the same object	Time duration	22.6 (*13.2*)	28.0 (*20.8*)	−1.07	*0.292*
	Frequency	70.0 (*53.8*)	84.5 (*62.3*)	−0.88	*0.386*
Infant sustained attention to the target object	Time duration	112.5 (*56.4*)	108.6 (*43.0*)	0.28	*0.782*
	Frequency	21.9 (*9.7*)	20.7 (*6.1*)	0.51	*0.612*

As for social coordination, we examined four measures: (1) all phrase use while the infant looked at an object, (2) specific phrases containing the target label while the infant looked at the target object, (3) presence of an optimal naming moment, and (4) joint attention between the parent and infant toward the same target object. Optimal naming moment refers to an instance when parent object labeling occurs while the infant is simultaneously looking at the target object, and over 70% of the object is captured by the infant’s egocentric view. Joint attention here refers to the moment where the parent and infant both attended the same object. Both the dominance of the target object in the infant’s visual field during the naming moment (Pereira et al., 2014; Slone et al., 2019) and joint attention (Markus et al., 2000; Morales et al., 2000) have been shown to predict the infant’s effective sustained attention to a referent object and later word acquisition. The present study analyzed the potential language group differences by both the frequency and duration measures.

Given the substantial amount of data points expected per dyad, we also applied sequential path analysis to test the role of social input on guiding infant sustained attention to the referent items. The use of a large number of data points from a small number of dyads is consistent with previous studies considering multisensory systems using similar approaches and technologies for the study of parental references (e.g., Yu and Smith, 2012; Yoshida et al., 2020). Bootstrapping was used to randomly resample from the existing data points and to demonstrate the consistency of the estimates and model convergence.

## Results

### Parent Responsiveness

#### Parent’s Gaze Allocation

A series of MANCOVA with age and SES as covariates in both the measures of duration and frequency on the target behaviors were used to determine the effect of the language group. First, a MANCOVA in the duration of parent’s attentional preference for each of the four ROIs found no effect of language group but a marginal age effect, *F*(4, 40) = 0.20, *p* = 0.058. The follow-up univariate analysis for each of the four ROIs, using a Bonferroni-adjusted alpha level of 0.0125, revealed a developmental change in the duration of attention on the infant’s hands: parents took more time looking at the infant’s hands as the infant became older, *F*(1, 43) = 7.82, *p* = 0.008. Similarly, another MANCOVA in the frequency of the four ROIs found a significant change by age, *F*(4, 40) = 6.38, *p* < 0.001. Specifically, parents had more frequency of attention to target objects, *F*(1, 43) = 9.89, *p* = 0.003, and to the infant’s hands, *F*(1, 43) = 8.89, *p* = 0.004.

#### Verbal Input

**Figure 2:**
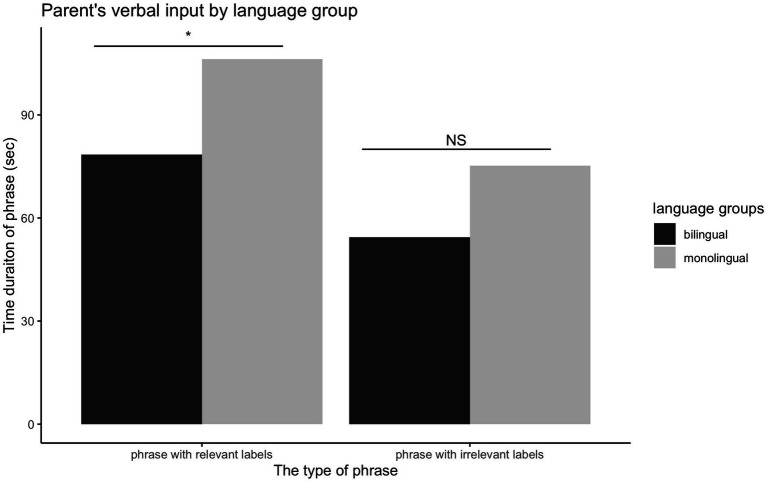
The distribution of parent’s verbal input by cultural groups.

Parental verbal input was analyzed as the number of phrases and target words used in the play session. First, parents in the monolingual group spoke more to their infants, as reflected in the duration (*t*(49) = 3.08, *p* = 0.004), as well as the frequency of phrases, *t*(49) = 2.81, *p* = 0.007. A MANCOVA with age and SES as covariates was performed to examine the difference between language groups on the duration of phrases, which were categorized by relevant or irrelevant labels. There was a significant effect of language group on phrases, *F*(2, 42) = 3.45, *p* = 0.041. The follow-up univariate analyses, using a Bonferroni-adjusted alpha level of 0.025, showed that parents in the monolingual group had longer phrases containing relevant labels, *F*(1, 43) = 4.55, *p* = 0.039, but the group difference was absent for phrase containing irrelevant labels, *F*(1, 43) = 2.31, *p* = 0.136 (see Figure 2). On the other hand, the MANCOVA model on the frequency of phrases showed no difference between language groups, *F*(2, 42) = 2.80, *p* = 0.072. In addition to parent’s phrases, we found that parents in the monolingual group used more labels on the target objects than did parents in the bilingual group, *t*(49) = 3.08, *p* = 0.003. These findings indicate that parents in the monolingual group provided more relevant verbal input that corresponded to referent toy objects during the play session, but no difference was found for irrelevant verbal input.

### Infant’s Looking Behaviors

Corresponding to findings from previous head-camera studies, infants from both language groups spent the majority of the time looking at the target objects, which accounted for an average of 47.04% (SD = 17%) of the time during the play session (e.g., Yoshida and Smith, 2008; Yu and Smith, 2013, 2017). MANCOVA analyses with age and SES as covariates were performed on both measures of duration and frequency of infant attention toward the four ROIs. There was no significant effect of language group on infant attention as reflected in either frequency or duration. The model for frequency found a developmental change over time: the older infants had higher attention counts on the four ROIs overall, *F*(4, 40) = 2.92, *p* = 0.032. Follow-up univariate analysis showed that infants allocated significantly more attention to the parent’s hands over time, *F*(1, 43) = 7.28, *p* = 0.009. Combined with the distribution of the parent’s attention, these findings imply that social coordination between the parent and the infant is established primarily by attending to the action of parent object handling.

### Social Coordination and Learning Pathways to Sustained Attention

Social coordination between parent responsiveness and infant attention was measured by four variables: (1) all phrase use while the infant looked at an object, (2) phrases containing the target label while the infant looked at the target object, (3) presence of an optimal naming moment, and (4) joint attention toward the same target object. The MANCOVA model for the frequency of the social coordination behaviors, with age and SES as covariates, indicated a significant language group effect: the monolingual cultural group had more social coordination overall, *F*(4, 40) = 3.27, *p* = 0.021. Follow-up analyses for each of the four types of social coordination showed that the monolingual group had more phrases use while the infant looked at the objects [*F*(1, 43) = 12.79, *p* < 0.001], more phrases containing labels for the infant-attended object [*F*(1, 43) = 10.45, *p* = 0.002], and more optimal naming moments, *F*(1, 43) = 7.12, *p* = 0.011. There were no group differences but a marginal age effect on the frequency of joint attention though, *F*(1, 43) = 3.96, *p* = 0.053.

Considering the frequency of the socially coordinated moments, especially the optimal naming moment, could be biased according to the transcription criteria (e.g., the length of the phrases, the frequency of the target words in phrases), we also examined the effect of language group in social coordination in terms of time duration. The MANCOVA model with age and SES as covariates showed no language group effect in the amounts of social coordination overall, *F*(4, 40) = 1.71, *p* = 0.197. In specific, Follow-up analyses for each of the four types of social coordination demonstrated that infants in both the monolingual and bilingual cultural groups had comparable amounts of time in attending to the referent object when parent talked [*F*(1, 43) = 1.32, *p* = 0.256], or when parent named the object simultaneously, [*F*(1, 43) = 1.51, *p* = 0.226]. Similarly, both monolingual and bilingual cultural groups experienced similar amounts of time in optimal naming moments [*F*(1, 43) = 1.78, *p* = 0.189] and in joint attention, *F*(1, 43) = 1.10, *p* = 0.300.

**Figure 3:**
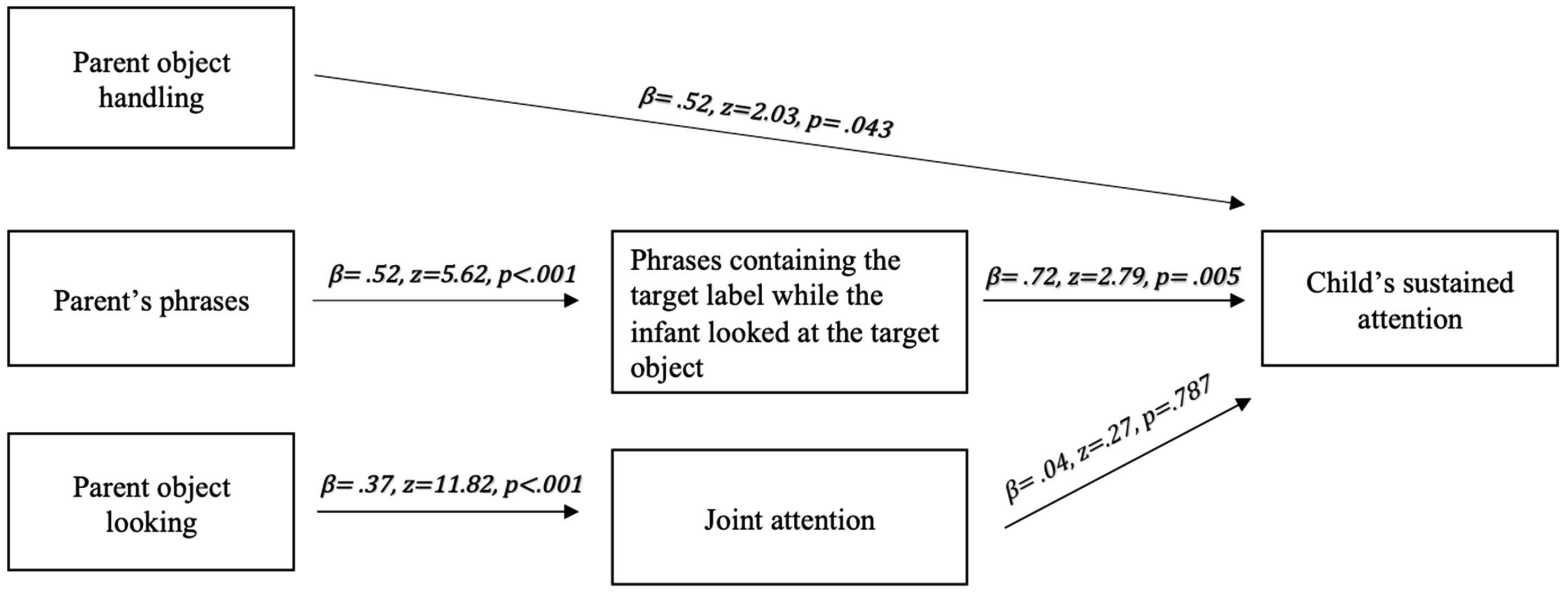
The sequential path analysis of parental referential input on child’s object looking through social coordination.

To fully characterize the various referent-driven pathways directing the infant’s multimodal experience and contributing to the infant’s sustained attention, a sequential path analysis was conducted. Sustained attention to the target object was defined as consistent gaze fixation over 2 s. The long fixation ensures that the infant has stable attention toward the referent item for further word learning. The left side of the sequential path includes three potential referential inputs from parent involvement during the play session: (1) object handling, (2) phrases, and (3) object looking. We added a multiple regression on each input variable, and its beta weights were also added as the path weight on the structural model shown in Figure 3. The sequential path analysis reveals two significant socially coordinated moments between parental input and infant attention across the language learning groups: (1) parent’s phrase while infant looked at the object, and (2) parent’s object handling while infant looked at the object. On the one hand, parental verbal input predicts infant sustained attention when the phrase contains the target label while the infant is simultaneously looking at the target object, *β* = 0.72, *Z* = 2.79, *p* = 0.005. On the other hand, parent object looking predicts joint attention between the parent and the infant on the target object (*β* = 0.37, Z = 11.82, *p* < 0.001), whereas joint attention does not contribute to infant sustained attention as expected, *β* = 0.04, *Z* = 0.27, *p* = 0.787. In addition, parent object handling also shows as a direct predictor of infant sustained attention, *β* = 0.51, *Z* = 2.03, *p* = 0.043.

## Discussion

Children’s language development has often been measured by speech input and language outcomes—what they hear in their social environment and what they can understand and produce (receptive and productive vocabularies). The literature on bilingual children’s language development suggests the importance of the quantity and quality of linguistic input (Rowe, 2012; Hoff et al., 2014; Unsworth, 2016; Carroll, 2017; De Houwer et al., 2018) and reveals a close relationship between parent’s verbal input and children’s vocabulary outcomes (De Houwer, 2007, 2011; Cartmill et al., 2013; Hoff and Core, 2013; Sorenson Duncan and Paradis, 2020). However, we know relatively little about early language experiences as a series of socially coordinated events from the child’s perspective. This is a critical gap in the literature given the increasing attention to the importance of multimodal language exposures that are created socially. The present study documents *dynamic multimodal experiences* that are supported by the parent’s scaffolding in parent–infant interactions.

Three significant findings characterize the early multimodal experiences that are relevant to bilingual language development. First, we found reliable differences between monolingual and bilingual groups in the amount of parental linguistic input. Based on the total number of phrases spoken, regardless of the specific languages used by the bilingual group, parents in the monolingual group spoke more and had more relevant labels for the referent items during the play session. This finding appears to echo the difference in the verbal input between children who are growing up from monolingual and bilingual households (Place and Hoff, 2011; Thordardottir, 2011; Hoff and Core, 2013; Hoff et al., 2014). These quantitative differences in language input are often discussed with respect to SES factors, which include parental education, annual household income, governmental assistance, and so forth (Scheele et al., 2010; Cartmill et al., 2013; Calvo and Bialystok, 2014; Hoff et al., 2018; Masek et al., 2021). Despite no language group difference being found on SES measures, we noticed that more monolingual parents in the present study had an annual income greater than $100,000 while bilingual parents had income within the range from $50,000 to $100,000 (see the distribution of annual household income in the Supplementary Material). Considering the variations between language groups and within the bilingual dyads, the present study added SES as a covariate in the analyses and yielded no SES effect on the target behaviors. Yet, caution is still needed in drawing conclusions about the causes of variations in parental verbal input. The further study aims to reach a larger sample size to systematically address these potential effects and fully capture the language experiences in diverse linguistic/sociocultural bilingual populations (e.g., across various countries and cultures).

What else might be the reason for the reduced verbal input in the bilingual group? There are two conjectures, one of which concerns a differential effect of the laboratory experience. Despite efforts to make the laboratory experience similar to typical interactive contexts as homes, the laboratory setting is novel, and the parent–infant linguistic interaction in the laboratory cannot be regarded as an exact replication of home interactions. Consequently, one may argue that the reduced linguistic experiences could be due to that “lab effect” (*viz*., to be observed in the semi-structural lab environment). Perhaps this lab effect, such as the novelty of people in the lab, room arrangement, and atmosphere, influences participants in the bilingual group more than the monolingual group. Further, though we asked our participants to freely use their preferred language during their visits, including the play session, they may perceive an implicit pressure to prefer English outside of their homes, resulting in less speech in the recording.

Most of the observational findings in the literature on language learning are based on learning contexts in which parents use English only at home, but language learning is more dynamic and complex for young children growing up in bilingual environments. We do not have any means of assessing the suitability of the observation method for each language group, yet this is something we need to take into consideration when we draw conclusions about differences in verbal input. In future studies, researchers could ask parents to complete a survey for evaluating the degree to which the activities in the lab were comfortable to the parents and if their performance felt typical and natural.

The second conjecture about differences in verbal input is that quantitative measures alone might be misleading. Insufficient and unbalanced language input in L1 and L2 has often been speculated to be responsible for developmental and linguistic differences, and this assumption has led to an increasing effort to supplement young children’s linguistic resources during preschool and elementary school periods (e.g., Winsler et al., 1999; Hammer et al., 2007; Collins, 2014). The present study, however, suggests that despite the less speech input, young children in the bilingual group are receiving similar amounts of optimal experiences that are well coordinated by parental scaffolding (in particular with duration measures), ensuring bilingual children have sufficient time mapping the heard words to the referent objects during their interaction with parents. The present findings show that infants from both monolingual and bilingual groups experienced a similar amount of joint attention—infant and parent sharing attention on the same target referent—that has been shown to promote language learning among young children in a monolingual environment (Markus et al., 2000; Morales et al., 2000; Akhtar and Gernsbacher, 2007). Although monolingual parents talked more and labeled the target items more frequently than the bilingual parents did, bilingual infants experienced comparable time in the coordinated moments during which parents timed their labeling of object names to coincide with the infant’s attention to the referent object. This is interesting, given that bilingual language research has heavily relied on large-scale measures and parental self-reports on some properties of linguistic quantity (e.g., De Houwer, 2007, 2011; Scheele et al., 2010; Byers-Heinlein, 2013; MacLeod et al., 2013) while overlooking variations in input quality as well as language learning processes (see reviews on quantity and quality input from Hoff and Core, 2013 and Unsworth, 2016). The present findings suggest that the overall amount of verbal input may be comparatively reduced, depending on the specific linguistic context, but the referent-related qualitative input remains constant. This assumption is corroborated by other studies, indicating that the quality of early parent input is not restricted by SES (Cartmill et al., 2013; Anderson et al., 2021). The measures on the qualitative input open a new window onto language measurements assessing learning growth in bilingual research.

Besides the word and phrase counts of verbal input, the literature on bilingual language development also points out several qualitative aspects of the input and variability in how each language is used. For example, the relative frequency of input in each of the two languages, the amounts of available resources (e.g., home only or instruction/academic purposes), the exposure structure (one-person-one-language vs. one-person-multiple-languages), and other variations in the experience of each language may influence the learning trajectories (Place and Hoff, 2011; MacLeod et al., 2013; Hoff et al., 2014; Unsworth et al., 2019; Orena et al., 2020). In the present study, we found that parents in the bilingual group used one language only and seldom switched languages in the play session. Other evidence also indicates that bilingual parents have a very low rate of language mixing before the infant’s age of 18 months (e.g., Meng and Miyamoto, 2012; Kremin et al., 2021). In addition, a bilingual parent’s language use might not be fully expressed in the limited time of the play session. Moreover, the bilingual families might employ a one-parent-one-language approach, in which case the infant would be exposed to another language only by other caregivers at home. Therefore, there is much room for sociocultural factors to explain the group difference in parental verbal input; additional studies of parent–infant interaction across different settings and social partners are necessary to identify those suspected factors.

The second major finding of the present study, in addition to the group difference in verbal input, is that parents support infant attention *via multimodal coordinated pathways* for both monolingual and bilingual groups. In our sequential path analysis of parental referential input, we observed social coordination that facilitates infant object looking. Specifically, the parent’s object naming events support infant object looking *via* the coordinated moments when the infant experiences “seeing an object while hearing the object name,” and parent object handling also supports infant attention to the referent object. Parent’s object labeling and handling can support the infant’s learning by helping the infant to recognize objects visually and to learn object characteristics, which may increase the likelihood of word–referent associations and promote further word learning (Gogate et al., 2006; Yu and Smith, 2012; Yu et al., 2019). Despite the fact that joint attention between the parent and the child toward the same object has been shown to predict the language, cognitive, and social development of monolingual children (e.g., Markus et al., 2000; Morales et al., 2000; Vaughan Van Hecke et al., 2007), we found the minimal impact of joint attention on infant’s object looking in the present study. It is speculated that gaze following and the establishment of gaze sharing can be more challenging in the daily interactive context, where infants may experience more complex visual environments consisting of multiple and colorful distractors.

Other studies of the infant’s visual input in the language development domain document the importance of social engagement and scaffolding in infant object looking, but most of those studies involve typically developing children in a monolingual culture in which parents use only English at home. The present study focuses on two different learning environments, English monolingual and diverse bilingual, and demonstrates important differences and similarities in the process through which infants find the referent of a name. Mapping names to referents is a core process of early language learning, that depends not only on linguistic input but also on how the verbal input is coordinated with perceptual experiences through social scaffolding. This line of work should be extended to a broader population whose learning contexts are different from those represented in the present study, and such initiatives have been taken. For example, in a similarly structured observational study using head-mounted camera devices, young children with Autism elicited an elevated level of parental responsiveness, such as parents looking at the infant’s face more often than parents of typically developing children (Yoshida et al., 2020). This finding corroborates the social coordination of object looking events found in both language groups participating in the present study.

The third important finding is that infants in the bilingual group obtain the same duration of item-relevant attention opportunities—optimal naming moments and joint attention—despite the reduced verbal input. While this might be due to elevated parental effort, one may speculate that being exposed to two languages from birth influences the infant’s speech perception and cognitive skills that underlie word learning (Kovács and Mehler, 2009a,b; Sebastián-Gallés et al., 2012; Brito and Barr, 2014; Comishen et al., 2019; Arredondo et al., 2022; Kalashnikova and Carreiras, 2022). Through persistent exposure to dual language and regularities specific to the contexts in which language is used, bilingual infants demonstrate enhanced flexibilities and sensitivities to the visual cues which help discriminate languages (Kovács and Mehler, 2009b; Sebastián-Gallés et al., 2012; Fort et al., 2018; Singh et al., 2018). For instance, infants from monolingual and bilingual environments show similar attention preference to a partner’s eye gaze in the preverbal period, whereas bilingual infants initiate attention shifting earlier and become more sensitive to the partner’s mouth movement, which has been demonstrated to be essential for word learning (Colunga et al., 2012; Pons et al., 2015; Tsang et al., 2018). Yow et al. (2017) also found that bilingual infants attend more to visual references, as indicated by eye gaze and pointing, in a fast-mapping task. In addition, other researchers have employed experimental methods to show advanced cognitive skills in bilingual infants even before word production (e.g., Kovács and Mehler, 2009a; D’Souza et al., 2020). Taken together, these findings suggest that bilingual environments, by themselves, may induce more attention shifting and a more concentrated focus of attention on specific stimuli or specific locations in experimental contexts. However, these differences in attentional behaviors are rarely reported in studies that are conducted in more naturalistic interactive environments. Attentional biases in bilingual children may shape parental responsiveness so as to generate a unique feedback loop that enhances the infant’s sensitivity to social cues and learning. To test this conjecture, further bilingual studies should consider differences in visual gaze patterns and attentional flexibility in relation to parental scaffolding across different settings, such as homes and daycares.

There are limitations in the present study that need to be taken into consideration for future research. First, we recognized the variabilities within the collected bilingual sample that may influence the degrees of parent’s engagements or the ways parents communicate with their infants. Infants growing up from various linguistic and sociocultural backgrounds may learn L1 and L2 at various growth rates, orient attention following parent’s responsiveness differently, and eventually develop diverse cognitive competence (e.g., Barac and Bialystok, 2012; Tran et al., 2019; Masek et al., 2021). Our goal of the present study was to investigate the impacts of early bilingual learning experiences on moment-to-moment multimodal experiences—looking and hearing—in an interactive social context. To address the concerns on the potential lab effect, further studies will implement this free-viewing paradigm into more naturalistic settings, such as home environment, and will systematically control cross-linguistic and cross-cultural variations to secure reliable homogeneous samples and validate the documented effect to broader bilingual populations.

Second, because children were recruited from a longitudinal research project, the age range (6–18 months) was relatively broad and included a transition period during which infants start to learn and produce words rapidly. Considering the dynamics of word acquisition and lexical development, parents might differ in the ways they respond, and the amounts of verbal input used (e.g., Soderstrom, 2007; Anderson et al., 2021). Given the limited sample size, we controlled for the infant’s age when comparing the language group differences. Ideally, we would examine any developmental changes in parents’ behaviors and infants’ subsequent attentional patterns in the near future.

Furthermore, the present study categorized parent’s verbal input according to their use of target words within phrases. In reality, parents may frequently use non-target words, such as “look,” “what is that,” and guide infant attention by pointing and/or through the use of gestures (Woodward and Guajardo, 2002; Deák et al., 2018). Further studies might benefit from a deeper analysis of the semantic content of parent verbal input and subtle ways in which it might guide infant attention and associated learning.

## Conclusion

The present study views “bilingualism” through a sociocultural lens and defines direct *input* operationally as the integrated and multimodal experiences of young children from monolingual and bilingual learning contexts. The study shows how similar social scaffolding shapes moment-to-moment attention for infants who have had different language experiences. Regardless of the total amount of parental speech input (how many words the infant hears), infants with monolingual and bilingual exposure both benefit equally from a comparable duration of name–object associations. These findings add to the current literature by demonstrating that the benefits of everyday language experiences are determined not only by linguistic input but also by multimodal experiences—coordinated visual and linguistic experiences—for children growing up in either a monolingual or a bilingual home environment.

## Data Availability Statement

The raw data supporting the conclusions of this article are not publicly available due to confidentiality but are available from the corresponding author on reasonable request.

## Ethics Statement

The studies involving human participants were reviewed and approved by IRB at University of Houston. Written informed consent to participate in this study was provided by the participants’ legal guardian/next of kin.

## Author Contributions

LS and HY contributed to the study design and data collection. LS organized the database and performed the statistical analysis and wrote the first draft of the manuscript. HY wrote sections of the manuscript. LS, CG, and HY contributed to manuscript revision, read, and approved the submitted version.

## Conflict of Interest

The authors declare that the research was conducted in the absence of any commercial or financial relationships that could be construed as a potential conflict of interest.

## Publisher’s Note

All claims expressed in this article are solely those of the authors and do not necessarily represent those of their affiliated organizations, or those of the publisher, the editors and the reviewers. Any product that may be evaluated in this article, or claim that may be made by its manufacturer, is not guaranteed or endorsed by the publisher.
