# Supplementary material for: Shared Multimodal Input Through Social Coordination: Infants With Monolingual and Bilingual Learning Experiences
Source: Front Psychol. 2022 Apr 19;13:745904. doi: 10.3389/fpsyg.2022.745904 (PMC9066094; doi:10.3389/fpsyg.2022.745904)
Supplement: Supplementary file 1 [file Data_Sheet_1.docx]

**Appendix**


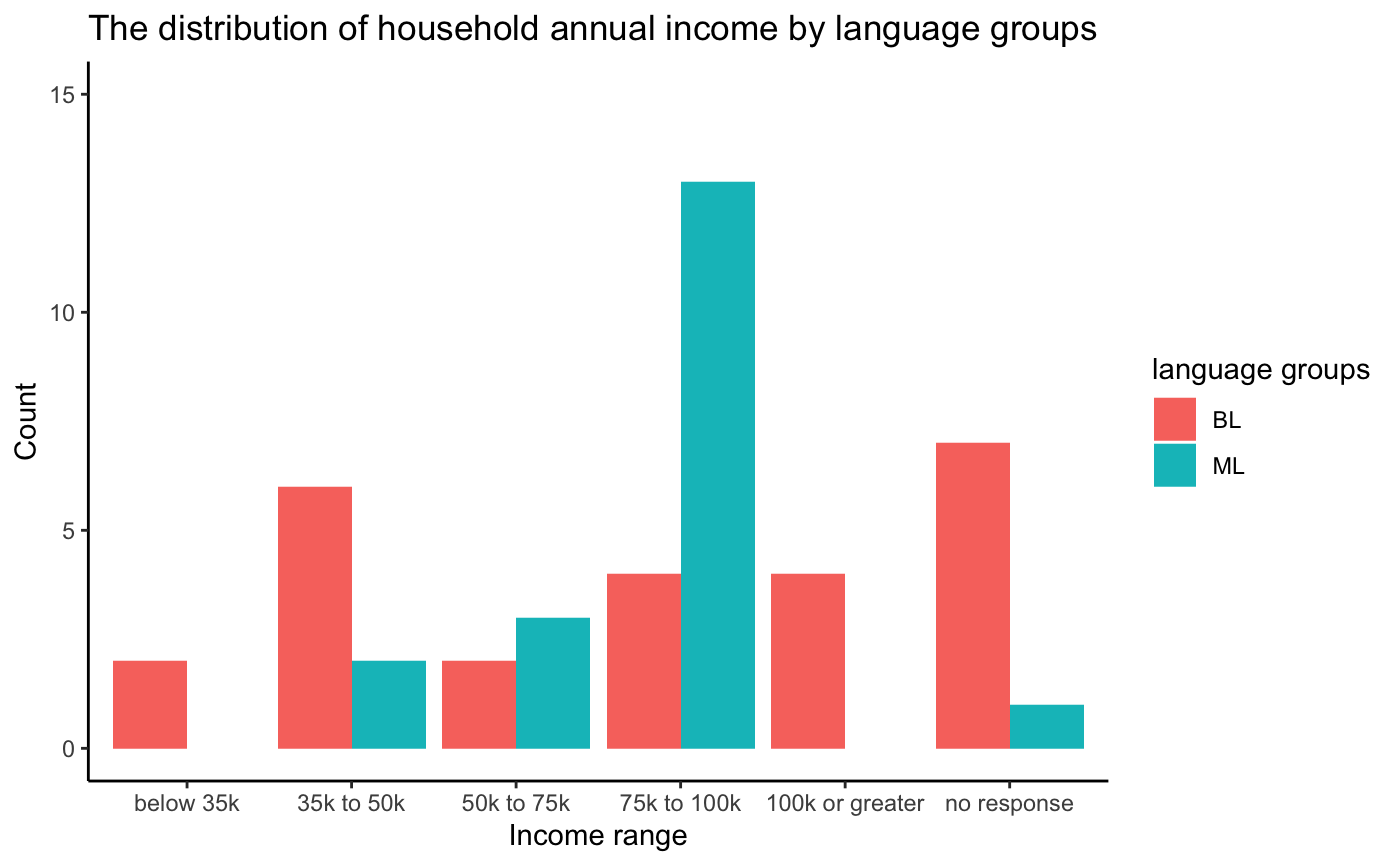


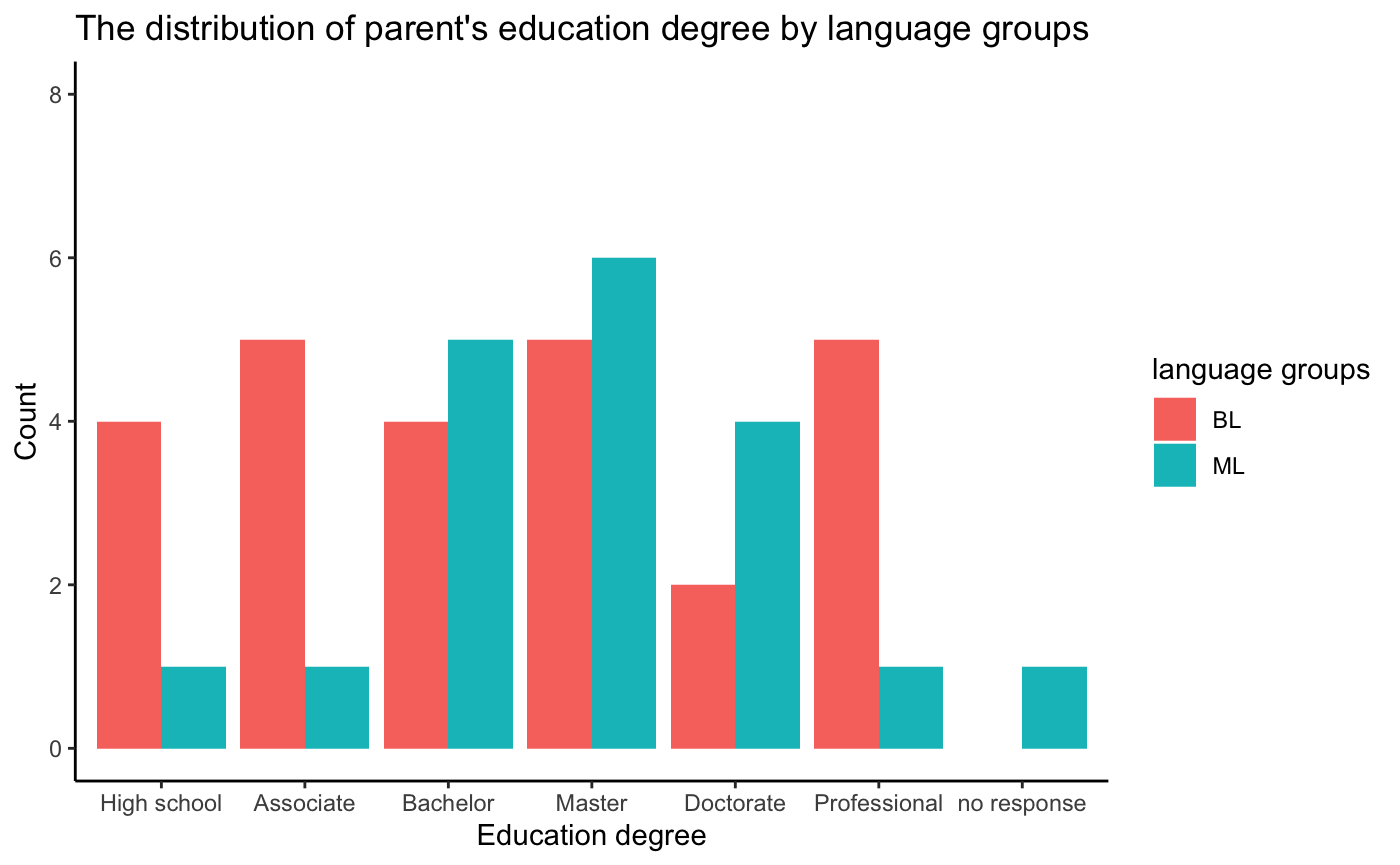


Table. Parent’s language usage in the play session

|  | **Language type by questionnaire** | **Audio instruction** | **language in the play session** | **Transcription** | **# Phrases has been translated** | **# Phrases has been transcribed** |
| --- | --- | --- | --- | --- | --- | --- |
| 1 | English, Spanish | English | English, Spanish | Two phrases in Spanish | 2 | 46 |
| 2 | English, Spanish | English | English only | English only | 0 | 82 |
| 3 | English, Spanish | English | English, Spanish | One phrase in Spanish | 1 | 114 |
| 4 | English, Spanish | English | English, Spanish | Five phrases in Spanish | 5 | 75 |
| 5 | English, Spanish | English | English only | English only | 0 | 142 |
| 6 | English, Spanish | English | English, Spanish | Nine phrases in Spanish | 9 | 148 |
| 7 | English, Spanish | English | Spanish, English | Thirty-one phrases in English | 230 | 261 |
| 8 | English, Spanish | English | English, Spanish | One phrase in Spanish | 1 | 95 |
| 9 | English and unidentified | English | English only | English only | 0 | 121 |
| 10 | English, Spanish | English | English, Spanish | one phrase in Spanish | 1 | 99 |
| 11 | English, Spanish | Spanish | Spanish only | Spanish only | 38 | 38 |
| 12 | English, Spanish | Spanish | Spanish only | Spanish only | 43 | 43 |
| 13 | English, Spanish | English | English only | English only | 0 | 27 |
| 14 | English, Spanish | English | English only | English only | 0 | 112 |
| 15 | English, Vietnamese | English | English, Vietnamese | One phrase in Vietnamese | 1 | 57 |
| 16 | English, Spanish |  | Spanish, English | Seven phrases in English | 7 | 54 |
| 17 | English, Krio | English | English only | English only | 0 | 41 |
| 18 | English, Chinese | English | English and Chinese | Fourteen phrases in Chinese | 14 | 49 |
| 19 | English and unidentified | English | English only | English only | 0 | 91 |
| 20 | English and Vietnamese | English | English only | English only | 0 | 97 |
| 21 | English and German | English | English only | English only | 0 | 145 |
| 22 | English and Tamil | English | Tamil and English | Nineteen phrases in English | 19 | 96 |
| 23 | English and Vietnamese | English | English only | English only | 0 | 127 |
| 24 | English, Spanish | Spanish | Spanish only | Spanish only | 127 | 127 |
| 25 | English, Spanish | English | Spanish and English | Fifty-one phrases in English | 68 | 119 |
| 26 | English, Spanish | English | English, Spanish | Nineteen phrases in Spanish | 19 | 134 |
| 27 | English, Spanish | English | English only | English only | 0 | 26 |
| 28 | English, Spanish | English | English only | English only | 0 | 120 |
| 29 | English, Chinese | English | English, Chinese | Six phrases in Chinese | 6 | 100 |
